# Gender difference in the associations of childhood maltreatment and non-suicidal self-injury among adolescents with mood disorders

**DOI:** 10.3389/fpsyt.2023.1162450

**Published:** 2023-05-25

**Authors:** Yan Yue, Yi Wang, Ruchang Yang, Feng Zhu, Xuna Yang, Xinchuan Lu, Ping Zhu, Zhengyan Wu, Zhe Li, Xueli Zhao, Xiangdong Du

**Affiliations:** ^1^Suzhou Guangji Hospital, The Affiliated Guangji Hospital of Soochow University, Suzhou, China; ^2^Suzhou Medical College of Soochow University, Suzhou, China

**Keywords:** non-suicidal self-injury, adolescent, childhood maltreatment, mood disorder, gender difference

## Abstract

**Background:**

Non-suicidal self-injury (NSSI) is a common feature among adolescents with mood disorders. Although childhood maltreatment has shown to be associated with non-suicidal self-injury (NSSI), previous studies have yielded mixed results in terms of different subtypes of childhood maltreatment and only few studies have investigated the effects of gender. The present cross-sectional study investigated effects of different types of childhood maltreatment on NSSI, as well as the role of gender in these effects.

**Methods:**

In this cross-sectional study, a total of 142 Chinese adolescent inpatients with mood disorders (37 males and 105 females) were consecutively recruited within a psychiatric hospital. Demographic and clinical characteristics were collected. Participants were administered the Childhood Trauma Questionnaire (CTQ), the Functional Assessment of Self-Mutilation (FASM).

**Results:**

76.8% of the sample reported engaging NSSI in the previous 12  months. Female participants were more likely to engage in NSSI than males (*p* < 0.001). Participants in the NSSI group reported significantly more experiences of emotional abuse (*p* < 0.001) and emotional neglect (*p* = 0.005). With regards to gender differences, female participants who have experienced emotional abuse were more likely to engage in NSSI (*p* = 0.03).

**Conclusion:**

As a whole, NSSI represents a frequent phenomenon among adolescent clinical populations and females were more likely to engage in NSSI than males. NSSI was significantly related to experiences of childhood maltreatment and specifically related to emotional abuse and emotional neglect over and above other types of childhood maltreatment. Females were more sensitive to emotional abuse than males. Our study highlights the importance of screening for subtypes of childhood maltreatment as well as considering the effects of gender.

## 1. Introduction

Non-suicidal self-injury (NSSI) is defined as the deliberate and direct destruction of one’s body tissue without suicidal intent and for purposes not socially or culturally sanctioned (1). Common forms of NSSI include cutting, biting, hitting, burning, and scratching (2). NSSI is most present among adolescents, with an estimated lifetime prevalence to be 17.2% for adolescents compared to 5.5% for adults in community samples (3). NSSI is even more prevalent in clinical samples and usually co-occurs with mood disorders (4, 5). It has been identified as a strong predictor of subsequent suicide behavior for adolescents and people who engage in NSSI are more likely to die by suicide (6–9).

Previous research has shown the link between childhood maltreatment and NSSI (10, 11). Across the globe, the overall estimated prevalence of common types of child maltreatment were 12.7% for sexual abuse, 22.6% for physical abuse, 36.3% for emotional abuse, 16.3% for physical neglect, and 18.4% for emotional neglect (12). There is substantial evidence that experiencing childhood maltreatment increases the risk of NSSI in adolescence. Previous research from both community and clinical adolescent samples has shown strong associations between childhood maltreatment and NSSI (13–16). However, there are differences in the relationships between specific types of childhood maltreatment and NSSI. On the one hand, sexual abuse has received considerable research attention and there is some evidence that sexual abuse is associated with an increased risk of NSSI relationship (17–19). On the other hand, previous studies have yielded mixed results for other types of childhood maltreatment (10, 11). Further research is needed in this field.

In terms of gender difference, not enough studies have investigated the moderating role of gender in the relationship between childhood maltreatment and NSSI. One study found that physical abuse and physical neglect increased the odds of NSSI among females, but for males, only physical abuse was associated with more NSSI behavior (20). Yet another study found no impact of physical abuse on NSSI for both genders (13). The discrepancy might be explained by the heterogeneity of the study cohorts. As a whole, the role of gender remained unclear due to a lack of studies.

Patients with mood disorders were more likely to have experiences of childhood maltreatment and more susceptible to NSSI. In a study of patients with major depressive disorder and bipolar disorder, Janiri et al. found that childhood emotional abuse, the severity of depression and female gender were associated with lifetime suicide attempts (21). Another study has shown that there was a significant association between emotional abuse score and the age of onset in drug-free bipolar depression patients (22). These studies have suggested the specific role of childhood maltreatment in the occurrence and progression of mood disorder.

In China, a recent study found that 62.2% of patients with depression or bipolar disorder reported NSSI in the past year (5). In addition, a systematic review estimated that 8.7% of Chinese children have suffered sexual abuse, 26.6% physical abuse, 19.6% emotional abuse and 26.0% neglect (23). Considering the high prevalence of both NSSI and childhood maltreatment in China and their potential clinical consequences, it is of great importance to explore their relationship in people with mood disorders.

To better understand the risk factors related to NSSI as well as informing clinical implications for prevention and intervention, the current study aimed to investigate the association between specific types of childhood maltreatment and NSSI. We examined this relationship in a clinical adolescent sample, including an exploration of gender differences. It was hypothesized that individuals with experiences of childhood maltreatment would be more likely to engage in NSSI and this relationship would be different for specific types of maltreatment. In addition, gender would moderate the relationship between childhood maltreatment and NSSI.

## 2. Methods

### 2.1. Participants

The participants for this study were consecutively admitted adolescents to a psychiatric inpatient unit in Suzhou GuangJi Hospital between Jan 2021 and Dec 2021. The sample consisted of 142 subjects, including 37 males and 105 females. All participants recruited in this study met the following inclusion criteria: (1) aged between 12 and 18 years; (2) diagnosed with major depressive disorder or bipolar disorder currently with a depressive episode by two independent experienced psychiatrists, according to the International Statistical Classification of Disease and Related Health Problems, 10th Edition (ICD-10); (3) able to understand and participate in the clinical assessment. Exclusion criteria included intellectual disability, autism spectrum disorder, organic brain syndrome and any psychotic disorders such as schizophrenia. Patients diagnosed with two or more mental disorders were also excluded.

After a full explanation of the study, written consent was obtained from all participants. This study was approved by the ethical committees of Suzhou GuangJi Hospital. All study procedures were carried out in Suzhou GuangJi Hospital.

### 2.2. Measures

#### 2.2.1. Demographic data

Basic demographic data included age, gender, years of education and residence. Clinical characteristics included age of onset, history of NSSI (yes, no).

#### 2.2.2. Clinical symptoms measures

##### 2.2.2.1. Non-suicidal self-injury

The Functional Assessment of Self-Mutilation (FASM) measures the methods and functions of NSSI. It is a self-report questionnaire to assess frequency of 11 NSSI behaviors and 22 function domains within the last 12 months (24). In the current study, we used the Chinese Version of FASM (C-FASM) (25). It includes a 10-item method checklist (e.g., cutting, scratching) and 15-item NSSI function checklist (e.g., to stop bad feelings; to punish yourself). Responses were rated on a scale of 1 (never) to 4 (very often). In the current study, we mainly used the method checklist and the Cronbach α coefficient of the method list was 0.78.

To avoid misunderstanding, researchers explained definition of NSSI to participants beforehand. Participants were told that: “self-injury means deliberately hurting yourself without the intent of killing yourself.” Participants were then asked if they had self-injured in the last 12 months. Data regarding methods, frequency, severity, and age at onset of NSSI behaviors was collected to ensure that they met the criteria of NSSI. In the current study, we used NSSI as a binary outcome for logistic regression analysis.

##### 2.2.2.2. Childhood maltreatment

Childhood maltreatment was assessed by the Child Trauma Questionnaire (26). It is a 28-item self-report questionnaire assessing the severity of five types of childhood maltreatment: emotional abuse, emotional neglect, physical abuse, physical neglect, and sexual abuse. Responses were rated on a scale of 1 (never true)- 5 (very often true). A higher score indicates more experiences of childhood maltreatment. The CTQ has demonstrated good reliability and validity among Chinese adolescents (27). With the present study, the Cronbach α coefficient as a whole was 0.73.

### 2.3. Data analysis

To compare demographic data and clinical characteristics, Chi-square test and student t test were applied for categorical variables and continuous data, respectively. A binary logistic regression analysis, with the “enter” method, was conducted to examine the relationship between specific types of childhood maltreatment and NSSI. The computation of separate models for male and female participants allowed for the examination of gender differences. To control for the confounding variables, age and psychiatric diagnosis were specified as covariates. The magnitudes of association between NSSI behavior and specific types of child maltreatment were based on odds ratio with 95% confidence intervals. These OR can be interpreted as a measurement of increase or decrease of NSSI in the presence of different types of child maltreatment. All tests were performed with the Statistical Package for the Social Science (SPSS) version 23.0. The *p*-values were set as two-tailed with the significance level α = 0.05.

## 3. Results

### 3.1. Social demographic and clinical characteristics of the sample

As shown in Table 1, with regards to demographic data, male participants were significantly older than female participants (*t* = 3.15, *p* = 0.002). There was no significant gender difference in any other social demographic characteristics including years of education and residence (both *p* > 0.05).

**Table 1:** Social demographic and clinical characteristics of the sample.

	Male (*n* = 37)	Female (*n* = 105)	*t*/*χ*^2^	*p*
Age	15.27 ± 1.67	14.32 ± 1.54	3.148	0.002^**^
Diagnosis				
Major depressive disorder	30(81.1)	89(84.8)	0.273	0.609
Bipolar disorder	7(18.9)	16(15.2)		
Age at onset				
	13.15 ± 2.83	12.82 ± 1.66	0.501	0.621
Years of education				
	9.05 ± 1.76	8.54 ± 1.58	1.64	0.104
Residence				
Rural	6	26	1.15	0.363
Urban	31	79		
NSSI				
Yes	20(54.1)	89(84.8)	14.462	<0.001^***^
No	17(45.9)	16(15.2)		
NSSI frequency				
	86.1 ± 22.8	121.7 ± 14.6	−1.27	0.206
CTQ subscale				
CTQ emotional abuse	10.8 ± 5.3	10.8 ± 4.6	0.041	0.967
CTQ physical abuse	7.8 ± 5.3	6.8 ± 3.2	1.471	0.143
CTQ sexual abuse	6.2 ± 3.3	6.5 ± 4.0	0.804	0.423
CTQ emotional neglect	13.1 ± 5.2	13.7 ± 4.6	−0.66	0.511
CTQ physical neglect	10.5 ± 3.8	9.0 ± 3.0	2.486	0.014*

Bold values meet significance at *P < 0.05, **P  < 0.01, and ***P  < 0.001.

With regards to clinical characteristics, a total of 109 (76.8%) participants reported 12-month engagement in NSSI. Twenty male participants (54.1%) and 89 female participants (84.8%) reported engagement in NSSI within the last 12 months. The proportion of female participants in the NSSI group was higher than that of males compared with the non-NSSI group (*χ*^2^ = 14.462, *p* < 0.001). In terms of types of childhood maltreatment, male participants were more likely to experience physical neglect than female participants (*t* = 2.486, *p* = 0.014). There was no significant gender difference in any other types of childhood maltreatment (all *p* > 0.05). In addition, no significant gender difference was identified in terms of age at onset (*p* > 0.05).

### 3.2. Gender differences of childhood maltreatment experience between participants with and without NSSI

As shown in Table 2. participants in the NSSI group reported having experienced more childhood maltreatment than the non-NSSI group (*t* = −2.85, *p* = 0.005). In particular, participants who have experienced emotional abuse and emotional neglect were more likely to engage in NSSI (*t* = −3.71, *p* < 0.001; *t* = −2.87, *p* = 0.005). There was no significant difference in terms of any other types of childhood maltreatment (all *p* > 0.05).

**Table 2:** CTQ scores of patients with and without NSSI.

	NSSI (*n* = 109)	No NSSI (*n* = 33)	*T*	*p*
CTQ total score	48.7 ± 14.2	41.0 ± 11.3	−2.853	0.005*
CTQ emotional abuse	11.6 ± 5.0	8.2 ± 2.9	−4.910	<0.001***
CTQ physical abuse	7.2 ± 4.0	6.5 ± 3.4	−0.879	0.381
CTQ sexual abuse	6.4 ± 3.6	5.5 ± 1.9	−1.773	0.079
CTQ emotional neglect	14.1 ± 4.9	11.5 ± 4.0	−2.871	<0.005**
CTQ physical neglect	9.4 ± 3.3	9.3 ± 3.3	−0.225	0.822

Bold values meet significance at *P < 0.05, **P < 0.01, and ***P < 0.001.

With regards to gender differences, as shown in Table 3, the result of a binary logistic regression showed that emotional abuse was a significant predictor of NSSI for females. For each additional point a female scored on emotional abuse scale, the odds of NSSI behavior increased by approximately 40%. However, there were no significant predictors for NSSI among male participants (All *p* > 0.05).

**Table 3:** Logistic regression of Non-suicidal Self-Injury (NSSI) on Childhood Trauma Questionnaire Subscale Scores.

	Male								Female							
	Incidents of NSSI (unadjusted)				Incidents of NSSI (adjusted)				Incidents of NSSI (unadjusted)				Incidents of NSSI (adjusted)			
Predictor	B	SE	OR	95%C.I.	B	SE	OR	95%C.I.	B	SE	OR	95%C.I.	B	SE	OR	95%C.I.
CTQ neglect	0.1	0.12	1.1	0.88–1.38	0.09	0.12	1.1	0.87–1.40	0.08	0.09	1.09	0.09–1.31	0.05	0.1	1.06	0.87–1.28
CTQ physical	–0.3	0.16	0.75	0.54–1.02	–0.3	0.16	0.76	0.56–1.04	–0.02	0.15	0.81	0.61–1.10	–0.2	0.16	0.8	0.59–1.10
CTQ emotional	0.32	0.17	1.38	1.00–1.90	0.25	0.17	1.28	0.92–1.78	0.33	0.15	1.40*	1.04–1.87	0.35	0.16	1.42*	1.04–1.94
CTQ sexual	0.42	0.37	1.52	0.74–3.11	0.39	0.32	1.47	0.79–2.75	0.06	0.14	1.06	0.81–1.38	0.05	0.14	1.05	0.81–1.37
CTQ physical	0.08	0.15	1.08	0.81–1.43	0.13	0.16	1.14	0.84–1.54	–0.2	0.13	0.79	0.61–1.03	–0.02	0.13	0.81	0.53–1.05

OR, odds ratio; C.l., confidence intervals; Bold values meet significance at *P < 0.05, **P < 0.01, and ***P < 0.001.

## 4. Discussion

The present study examined the relationship between NSSI and childhood maltreatment in an adolescent inpatient sample with mood disorders, including an exploration of gender differences. The major findings showed that childhood maltreatment predicted the risk of NSSI, and this relationship was different for specific types of maltreatment. Additionally, gender moderated the relationship between childhood maltreatment and NSSI.

The present study showed that in an adolescent inpatient sample with mood disorders, 76.8% reported engaging NSSI in the past 12 months. The rate appears a little higher compared to some previous studies (60–72%) regarding inpatient samples (17, 28, 29). This might be due to the fact we used FASM to measure NSSI which also includes minor forms of NSSI (e.g., Bit yourself). In addition, our inpatient sample included more females than males compared to previous studies, and these participants were diagnosed with either major depressive disorder or bipolar disorder Previous studies have shown that patients meeting NSSI disorder criteria were significantly more likely to be female and to be diagnosed with a mood disorder (30). Our results showed that female participants were more likely to engage in NSSI than their male counterparts. This finding is consistent with previous observations that NSSI is more frequently seen in females and that this disparity is generally greater in clinical populations (31–33). One explanation is that females were more dependent on NSSI to regulate their negative emotions (34, 35). Another possibility is that males are less likely than females to report NSSI (36).

The NSSI group scored significantly higher in the emotional abuse and neglect subscales of CTQ, suggesting that mood disorder patients with NSSI behaviors were more likely to have experiences of emotional abuse and neglect. There is some support for these relations (17, 37, 38). A recent meta-analysis (11) found pooled ORs ranged from small-to-medium effects for emotional neglect to medium-to-large for emotional abuse. With regards to the relation between emotional abuse and NSSI, Glassman and colleagues suggested that this relation is partially explained by the presence of self-criticism: Experiencing emotional abuse in one’s childhood may result in internalizing a negative critical thinking style toward the self and when these adolescents are faced with stressful events later in life, they are more likely to engage in NSSI for self-punishment (37).

In the present study, we found no statistically significant relationship between childhood sexual abuse and NSSI, which contradicted previous findings (17, 39, 40). This may be due to the low prevalence of sexual abuse in our sample rather than an indication of no relationship. Previous research showed that China has a lower incidence of sexual abuse than other countries (41). A meta-analysis showed that the prevalence of sexual abuse was 10.8% for Chinese women, which was lower than 19.7% for women in all countries, and for Chinese men, the rate was 4.8% compared to 7.9% globally (12). On the other hand, we did a further analysis showing that in those who engaged in NSSI, sexual abuse experience was positively correlated with NSSI frequency, suggesting that individuals may adopt NSSI as a coping strategy to sexual abuse.

There were some noteworthy gender differences. Emotional abuse seems to play an important role in NSSI behavior, especially for females. In unadjusted analysis, female patients with a history of NSSI behavior have significantly higher scores in the emotional abuse subscale, however, this was not found for males. This gender difference persisted even after adjusting for age and diagnosis. The results suggest that females are more sensitive to emotional abuse than their male counterparts, with a higher risk to result in NSSI behaviors. This finding is consistent with a previous study of clinical samples: Bernegger and colleagues showed in a clinical sample with mood disorders, emotional abuse was significantly associated with an increased odds ratio of NSSI for females but not for males (13). However, this finding is inconsistent with another previous study where researchers found little impact of emotional abuse on NSSI but physical abuse to be a significant risk factor for both genders (20). This might be due to the heterogeneity of the study cohorts. Our sample was recruited from inpatient clinics, which was similar to Bernegger’s sample, whereas Swannell and colleagues recruited participants from the general population (13, 20). This may suggest different underlying mechanisms of effects of childhood maltreatment on NSSI behaviors between clinical samples and the general population, and future research is needed to clarify these differences. The findings highlight the importance to examine separate effects of subtypes of childhood maltreatment on NSSI behaviors and gender should be considered as a moderating factor.

Results from the present study suggest that it is important to screen for childhood maltreatment history as it is useful to predict the risk of NSSI. Although overall childhood maltreatment is linked with NSSI, specific subtypes have different associations with NSSI and should be viewed independently. Despite these findings, there were several limitations in the current study. Firstly, this study was cross-sectional with a relatively small sample size, and longitudinal studies with larger sample size are needed in the future to better understand the temporal relationship between child maltreatment and NSSI. Another potential limitation was that we used retrospective self-report of child maltreatment which may suffer from the natural process of forgetting. There is also a likelihood that people with depression or bipolar disorder may have memory bias that their cognitive styles may lead to exaggerating past negative events (42–44). In the current study, we have attempted to reduce bias by clearly explaining the definition of child maltreatment to participants. Furthermore, adverse childhood experiences include many items other than childhood maltreatment such as parental separation and having family members in prison, whereas we did not include all these types in our study. Future studies may explore these items and to compare their effects on NSSI and mood disorders. Finally, our study subjects were inpatients, which may affect the prevalence of NSSI and the effects of childhood maltreatment. A healthy control cohort may help to compare the differences and improve the study.

Despite these limitations, our study highlights the importance of screening for subtypes of childhood maltreatment as it is a risk factor for NSSI in patients with mood disorders. In addition, clinicians should be aware of gender differences. Future research is needed to address the underlying mechanisms of gender difference.

## Data availability statement

The raw data supporting the conclusions of this article will be made available by the authors, without undue reservation.

## Ethics statement

The studies involving human participants were reviewed and approved by the Ethics committee of Suzhou GuangJi Hospital. Written informed consent to participate in this study was provided by the participants’ legal guardian/next of kin.

## Author contributions

XD and YY designed the study. RY, FZ, XY, XL, PZ, ZL, and XZ collected materials from study participants and conducted the analysis of all data. YW drafted the initial manuscript. XD, YY, ZW and ZL contributed to the revision of the manuscript. All authors read and approved the final manuscript.

## Funding

The study was funded by the Suzhou Gusu Health Talents Scientific Research Project (GSWS2021053, GSWS2019070), Key Diagnosis and treatment Program of Suzhou (LCZX202016), the Scientific and Technological Program of Suzhou (SKY2021062), and the Suzhou clinical Medical Center for mood disorders (Szlcyxzx202109).

## Conflict of interest

The authors declare that the research was conducted in the absence of any commercial or financial relationships that could be construed as a potential conflict of interest.

## Publisher’s note

All claims expressed in this article are solely those of the authors and do not necessarily represent those of their affiliated organizations, or those of the publisher, the editors and the reviewers. Any product that may be evaluated in this article, or claim that may be made by its manufacturer, is not guaranteed or endorsed by the publisher.
